# Multi-Level Thresholding Color Image Segmentation Using Modified Gray Wolf Optimizer

**DOI:** 10.3390/biomimetics9110700

**Published:** 2024-11-15

**Authors:** Pei Hu, Yibo Han, Zheng Zhang

**Affiliations:** School of Computer and Software, Nanyang Institute of Technology, Nanyang 473004, China

**Keywords:** multi-level thresholding, image segmentation, gray wolf optimizer

## Abstract

The success of image segmentation is mainly dependent on the optimal choice of thresholds. Compared to bi-level thresholding, multi-level thresholding is a more time-consuming process, so this paper utilizes the gray wolf optimizer (GWO) algorithm to address this issue and enhance accuracy. To acquire the optimal thresholds at different levels, we modify the GWO (MGWO) in terms of leader selection, position update, and mutation. We also use the Otsu method and Kapur entropy as objective functions. The performance of MGWO is compared with other color image segmentation algorithms on ten images from the BSD500 dataset in terms of objective values, variance, signal-to-noise ratio (PSNR), structural similarity index measure (SSIM), and feature similarity index (FSIM). Experimental and non-parametric statistical analyses demonstrate that MGWO performs excellently in the multi-level thresholding segmentation of color images.

## 1. Introduction

Image segmentation methods have received widespread attention in image recognition, early medical detection, etc. [1,2]. For optimal segmentation, the pixels within each region should have similar characteristics. Thresholding is a widely used image segmentation technique because of its simplicity, robustness, and accuracy [3,4]. Bi-level thresholding is a simple method that divides an image into two categories by searching for a single threshold. In contrast, multi-level thresholding divides an image into multiple parts.

In digital media today, color images hold more significance than grayscale images. Segmentation techniques for color images have gained increasing attention due to various specific applications in image content analysis and image understanding [5,6]. Color images combine different color channels (usually red, green, and blue, known as RGB). Each pixel is composed of various intensities of these three colors, resulting in rich colors and details. Color image segmentation is a method that partitions an image into different regions with unique properties and characteristics. Common color image segmentation methods include feature-based methods, image domain-based methods, and physics-based methods [7,8]. In the first category, cluster segments are homogeneous regarding feature space (such as intensity level, color, or texture). After mapping the pixels to the color space, they are assigned to clusters according to their features. In general, feature space techniques are spatially blind and ignore pixel color spatial distribution. Histogram thresholding techniques can be classified into the second category. Thresholding techniques divide pixels based on their intensity or color levels, which are connected to global or multiple thresholds. The histogram consists of relatively separated intensity parts, and each represents an object in an image. The third category, in which segmentation is performed by dividing data into a defined number of clusters, includes K-means and Fuzzy C-means algorithms [9].

There are two methods to handle optimal thresholding problems: parametric and non-parametric methods [10,11]. For parametric methods, it is assumed that the gray levels of each class follow a probability density function, and then, the statistical parameters for each class are calculated. Traditional non-parametric methods tend to find optimal thresholds for dividing gray regions based on criteria such as Renyi entropy, cross-entropy, and interclass variance (Otsu method). Thresholding techniques are suitable when the number of thresholds is small. However, as the number of thresholds increases, multi-level color image thresholding has a higher computational cost and requires an exhaustive search to find the optimal values.

To overcome these limitations, meta-heuristic methods [12,13], such as particle swarm optimization (PSO) [14,15], the salp swarm algorithm (SSA) [16], and the multi-verse optimizer (MVO) [17], have been applied to the field of image segmentation, and they have shown excellent performance. However, they often encounter the problem of local optimality [18,19]. We utilize the gray wolf optimizer (GWO) [20] for color image segmentation.

GWO selects leaders based on the wolves’ fitness values. However, in the modified GWO (MGWO), the leader selection process has been modified to enhance population diversity and avoid premature convergence. It improves the algorithm’s ability to explore the search space more effectively and increases the global search capability and the likelihood of finding the optimal segmentation threshold. MGWO introduces two mutations to acquire better solutions. The first mutation helps MGWO better capture complex image features and prevents the algorithm from making significant random jumps that could disrupt convergence. It improves the algorithm’s ability to fine-tune threshold levels during the segmentation process and thus leads to more precise results. The second mutation promotes population diversity and enhances the algorithm’s overall exploration capability. It is particularly valuable for complicated and high-dimensional problems, such as multi-level image segmentation, where the solution space may be large and irregular. Traditional thresholding techniques, such as the Otsu method and Kapur entropy, are usually applied to grayscale images. However, color images contain additional information (RGB channels). By optimizing multi-level thresholds for different color channels, MGWO provides more comprehensive segmentation that considers the complex relationships among the channels and improves the accuracy of the segmentation process. It is especially crucial in applications that require fine-grained segmentation to distinguish subtle variations in color intensity. The main contributions of this paper are as follows:

1. Establish a multi-level thresholding segmentation framework for color images.
2. Propose a GWO algorithm for image segmentation and improve it with the selection of leader wolves and mutation.
3. Validate the algorithm’s performance on the BSD500 benchmark dataset.

The main structure of this paper is as follows: Section 2 introduces the progress of related works in multi-level thresholding color image segmentation, and Section 3 explains the working principle of GWO and its modified version for solving multi-level thresholding. Section 4 covers the experimental analysis and presents a discussion of the results, and Section 5 presents the conclusions of this study and future research.

## 2. Related Works

Image segmentation is an important research topic in image processing. Over the past few decades, extensive work has been conducted, and multi-level thresholding segmentation based on meta-heuristic algorithms has been widely used due to its simplicity.

For the multi-threshold segmentation of grayscale and color images, the computational complexity increases exponentially with the number of threshold levels. Ma et al. introduced a new method for segmenting images that uses Kapur entropy as the objective function [21]. Horizontal and vertical crossover strategies are introduced in the bald eagle search (BES) algorithm. It is a high-quality image segmentation method in the multi-threshold segmentation of grayscale and color images. Guo et al. proposed a novel and efficient multi-level thresholding method for color images [22]. This method uses an energy function and incorporates the spatial context information of the images to generate energy curves. The proposed segmentation technique based on the energy curve uses inter-class variance, Tsallis entropy, and Kapur entropy as objective functions. It further utilizes the firefly algorithm (FA) to enhance segmentation performance. Anitha et al. proposed a modified whale optimization algorithm (MWOA) to optimize multi-level color image thresholding [23]. Otsu and Kapur have been utilized as fitness functions in the proposed algorithm. The cosine function is adjusted during the optimization process to control the positions in MWOA. Moreover, a correction factor is introduced in the position updates to regulate the movement of whales. These modifications of MWOA establish a suitable balance between exploration and exploitation and avoid local optima problems.

Xing employed the emperor penguin optimization (EPO) algorithm to determine the optimal multi-level thresholding for color images [24]. Gaussian mutation, Lévy flight, and adversarial learning are employed to enhance the search capabilities of the EPO algorithm. The experimental results demonstrate that this algorithm is a proficient approach to image segmentation. Moth–flame optimization (MFO) has a simple structure and strong selection ability. However, it is prone to falling into local optima and has slow convergence. Nguyen et al. proposed a new approach to improve MFO by incorporating Lévy flight and a logarithmic function into its update equations to improve the algorithm’s performance [25]. This algorithm is more efficient in multi-threshold image segmentation. PSO tends to experience premature convergence due to the loss of particle diversity in the latter stages. Dhal et al. aimed to solve the issues with the PSO algorithm and applied the improved algorithm in image segmentation [26]. They divide the population into multiple sub-populations through co-evolution, and the worst sub-population executes mutation based on probability. The proposed algorithm successfully segments blood cell images.

The number of thresholds affects the segmentation accuracy of color images. Wang et al. devised an SSA to select the ideal parameters and enhanced the SSA with Lévy flight [27]. During the optimization process, Kapur entropy, the Otsu method, and Renyi entropy are used to evaluate the solutions. Fu et al. proposed an efficient multi-level thresholding segmentation method based on the chimp optimization algorithm (IChOA) [28]. Kapur entropy is used as the objective function. IChOA is employed to identify the most suitable thresholds for the three channels of RGB images. In addition, population initialization is facilitated by the introduction of Gaussian chaos and opposition-based learning strategies. IChOA enhances population diversity and strengthens exploration and exploitation through these methods.

The above analysis demonstrates that the choice of thresholds significantly affects the accuracy and time required for image segmentation. Meta-heuristic algorithms can address this issue. Therefore, we use the GWO algorithm to perform multi-level thresholding segmentation of color images.

## 3. Multi-Level Thresholding Image Segmentation

**Figure 1:**
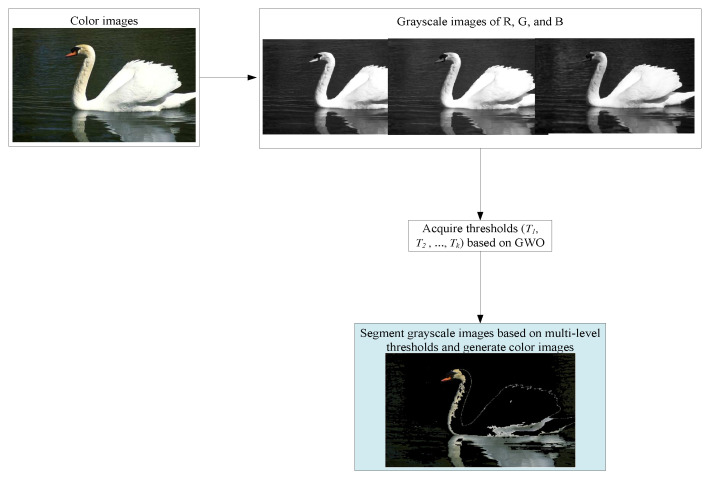
The segmentation process of color images.

Multi-level thresholding can accurately capture the details and complex structures in images and improve the segmentation effect. This technique is widely utilized in various fields, including medical image analysis, remote sensing image processing, and pattern recognition, and improves the accuracy of image analysis and the effectiveness of information extraction. For color images, we first extract their RGB channels and then segment each channel. Finally, the results of R, G, and B are combined for the final output. The specific process of color image segmentation is illustrated in Figure 1.

### 3.1. Objective Functions

In this study, we utilize the Otsu method and Kapur entropy as objective functions for multi-level thresholding image segmentation.

#### 3.1.1. Otsu Method

Otsu multi-threshold image segmentation is a statistical technique that maximizes the variance between classes. Suppose we want to divide an image into *K* categories, so we need to find K−1 thresholds $T_{1},T_{2},\ldots ,T_{K-1}$. The total between-class variance is defined as follows:

(1) $$\sigma_{b}^{2}=\sum_{k=0}^{K-1}\omega_{k}{\left(\mu_{k}-\mu_{T}\right)}^{2}$$

where $\mu_{T}$ is the overall mean gray level, and $\mu_{k}$ is the mean gray level of a class/region. Their equations are depicted in Equations (2) and (3).

(2) $$\mu_{T}=\sum_{i=0}^{L-1}i\cdot P\left(i\right)$$

(3) $$\mu_{k}=\frac{\sum_{i=T_{k-1}+1}^{T_{k}}i\cdot P\left(i\right)}{\omega_{k}}$$

where $\omega_{k}$ represents the weight of class *k*, *L* is the number of gray levels, and P(i) is the probability of gray level *i*.

(4) $$\omega_{k}=\sum_{i=T_{k-1}+1}^{T_{k}}P\left(i\right)$$

where $T_{0}=-1$ and $T_{K}=L-1$.

(5) $$P\left(i\right)=\frac{n_{i}}{N}$$

(6) $$N=\sum_{i=0}^{L-1}n_{i}$$

where $n_{i}$ is the number of pixels at gray level *i*.

#### 3.1.2. Kapur Entropy

The basic idea of Kapur entropy is to treat the histogram of an image as a probability distribution and find the best segmentation by maximizing the sum of the entropies of each segmented region. Kapur ensures that the segmented areas contain the most information possible.

For a combination of thresholds $T_{1},T_{2},\ldots ,T_{K-1}$, Kapur calculates the sum of the entropies of the segmented regions as follows.

(7) $$H=H_{1}+H_{2}+\ldots +H_{K}$$

(8) $$H_{1}=-\sum_{i=0}^{T_{1}}\frac{P(i)}{W_{1}}\log\left(\frac{P(i)}{W_{1}}\right)$$

(9) $$H_{k}=-\sum_{i=T_{k-1}+1}^{T_{k}}\frac{P(i)}{W_{k}}\log\left(\frac{P(i)}{W_{k}}\right)$$

(10) $$H_{K}=-\sum_{i=T_{K-1}+1}^{L-1}\frac{P(i)}{W_{K}}\log\left(\frac{P(i)}{W_{K}}\right)$$

(11) $$W_{1}=\sum_{i=0}^{T_{1}}P\left(i\right)$$

(12) $$W_{k}=\sum_{i=T_{k-1}+1}^{T_{k}}P\left(i\right)$$

(13) $$W_{1}=\sum_{i=T_{K-1}+1}^{L-1}P\left(i\right)$$

where $H_{1},H_{2},\ldots ,H_{K-1}$ are the entropies of the segmented regions.

### 3.2. Modified Gray Wolf Optimizer

The classic GWO algorithm uses three wolves, α, β, and γ, to lead the population search. Their update equations are defined in Equation (17) [29].

(14) $$X_{1}=X_{\alpha }-\left(2a\cdot r_{1}-a\right)*\left|2r_{2}\cdot X_{\alpha }-X_{i}\right|$$

(15) $$X_{2}=X_{\beta }-\left(2a\cdot r_{2}-a\right)*\left|2r_{2}\cdot X_{\beta }-X_{i}\right|$$

(16) $$X_{3}=X_{\gamma }-\left(2a\cdot r_{3}-a\right)*\left|2r_{2}\cdot X_{\gamma }-X_{i}\right|$$

(17) $$X_{i}=\frac{X_{1}+X_{2}+X_{3}}{3}$$

where $X_{i}$ represents the position of wolf *i*, and $r_{1}$, $r_{2}$, and $r_{3}$ are random values in the range of [0, 1]. *a* is a coefficient, and it linearly decreases from 2 to 0.

#### 3.2.1. The Leader Pool

The GWO algorithm relies on three leading wolves to guide the population search, and it has poor population diversity. The algorithm’s convergence rate will decrease if there are too many leaders. To overcome the limitations of the GWO algorithm, we propose a new approach for selecting leaders. A leader pool is established, and each leader is chosen randomly to lead the position update each time.

(18) $$X_{pool}=\left\{\alpha ,\beta ,\gamma ,\rho \right\}$$

The leader pool includes α, β, γ, and ρ, where ρ represents the opposite value of the leaders’ average positions.

**Figure 2:**
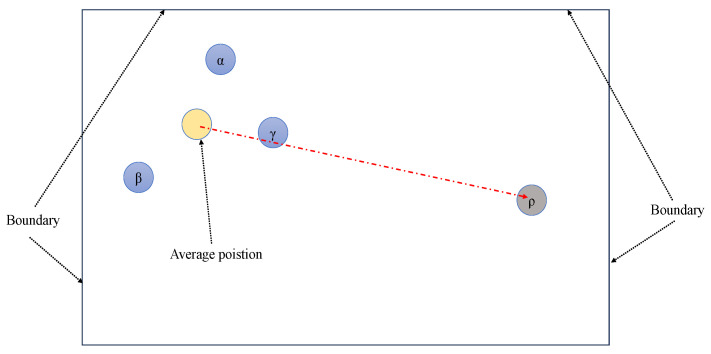
A demo of the leader pool (two-dimensional space as an example).

If α, β, and γ are close together, as shown in Figure 2, ρ can lead the population to search in broader areas and expand the search range. If α, β, and γ are far apart in the early stages of the algorithm, ρ also provides more learning directions. To increase the algorithm’s convergence, the probability of selecting a leader from the leader pool is different. α has a 50% chance of being selected, while the other leaders have an equal probability of being selected. The new update equation is modified as follows:

(19) $$X_{i}=X_{d}-\left(2a\cdot r_{1}-a\right)*\left|2r_{2}\cdot X_{d}-X_{i}\right|$$

where *d* is a number randomly selected from the leader pool.

This mechanism increases the possibility of the population obtaining more guidance information and alleviates the problem of insufficient population diversity. If wolves change their positions and discover that the new positions are not as good as their original positions, they will remain unchanged. This ensures that each wolf always stays in the best region it has explored, and improves the exploitation of the algorithm.

#### 3.2.2. Mutation

In multi-level thresholding image segmentation, the search space contains many local optimal solutions, and the space explored by wolves is much larger than their population size. Therefore, it is necessary to propose an improvement strategy to prevent the population from falling into local optima. The following mutation method is applied when a wolf does not update its position for five iterations.

1. The leaders

The leaders are responsible for guiding the population search, so their selection is crucial. The leaders need to mutate adaptively based on the stage of the algorithm. In multi-level thresholding image segmentation, the population’s dimensions are limited to 2, 3, 4, and 5, so the mutation dimensions of the leaders are at most two. The mutation amplitude of the leaders gradually decreases as the algorithm progresses.

(20) $$X_{i}^{j}=X_{i}^{j}+flag*round\left(rand\left(\right)*L*\left(MAXIT-it\right)/MAXIT\right)$$

where *j* means the dimension, and MAXIT and it represent the maximum and current iterations. flag is a random value in the range of {−1, 1}. round() and rand() represent the rounding function and the function for generating random numbers in the range of [0, 1].

This controlled mutation helps maintain diversity and encourages the exploration of new potential optimal regions.

2. The others

To enhance the global search performance of the algorithm, the remaining wolves update their positions using a variant of the opposition-based learning (OBL) method. The dimensions of the population are sorted in ascending order before executing the objective function in multi-level thresholding image segmentation, so their dimensions are not distinguished by order. We employ a random OBL method to update the positions to avoid scenarios where (2,252) could become (252,2) after OBL.

(21) $$X_{i}^{j}=\left(1+L\right)/2-round\left(rand\left(\right)*X_{i}^{j}\right)$$

**Figure 3:**
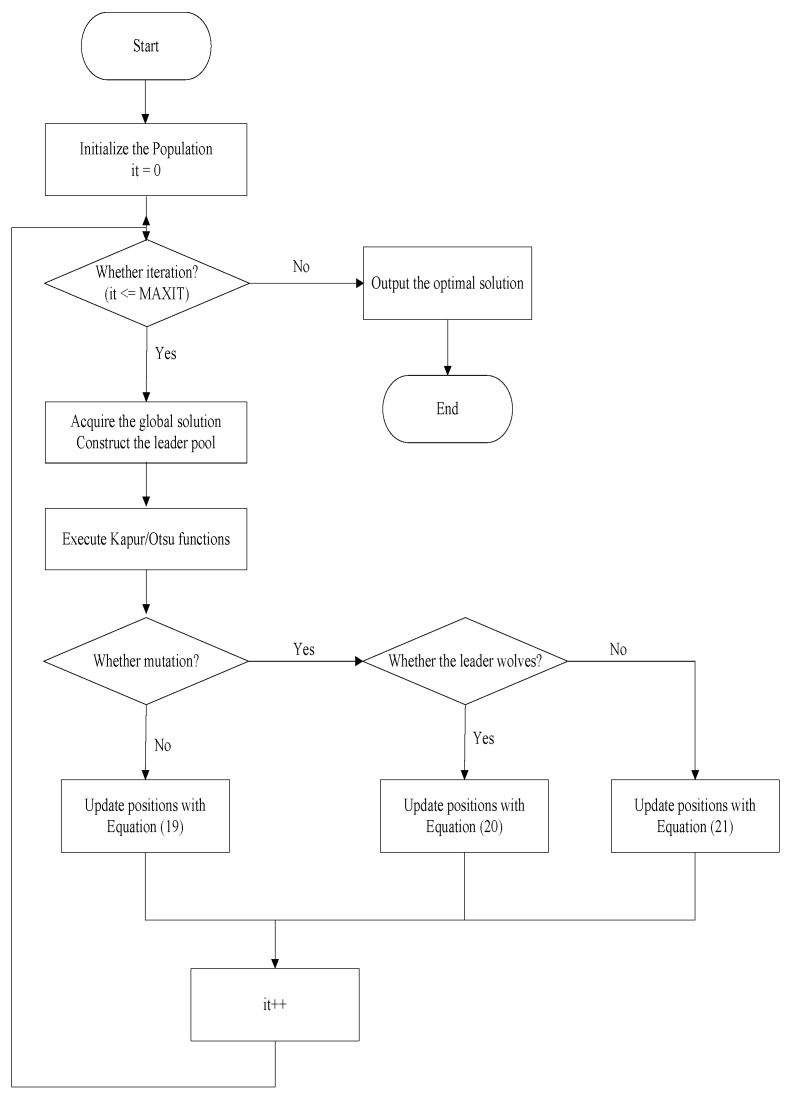
A flow chart of MGWO.

OBL effectively explores the search space, allowing the population to discover better solutions more rapidly and avoid local optimal solutions. A wolf randomly generates three candidate positions and selects the one closest to the currently sorted position as its next move. Figure 3 shows the flow chart of MGWO.

## 4. Experimental Results and Analysis

**Figure 4:**
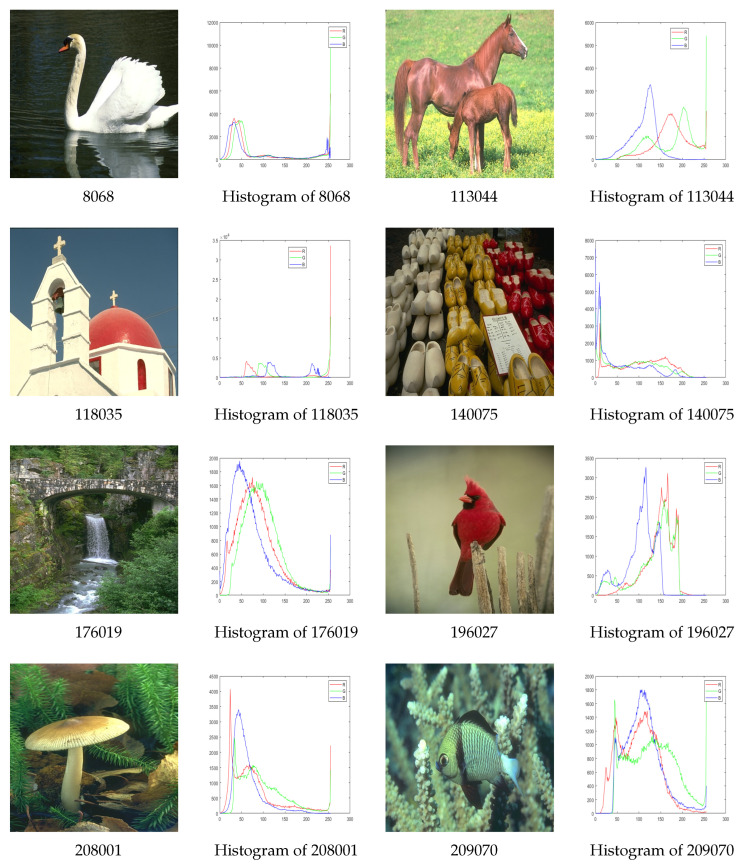

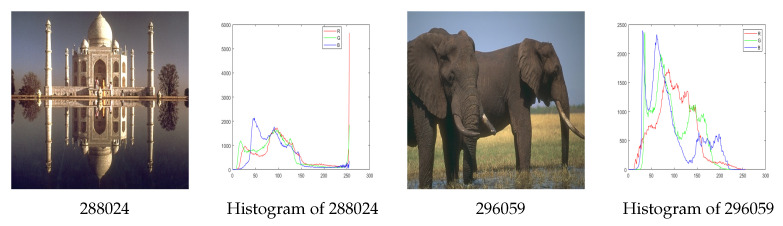
The test images and their histograms.

Ten images are randomly selected from the BSD500 benchmark [30] to evaluate segmentation performance, and we use two, three, four, and five threshold levels to maximize the objective functions for each test color image. All test images and their corresponding histograms are shown in Figure 4. The performance of the proposed MGWO algorithm is compared with GWO [20], MWOA [23], and FOA [31]. The BSD500 benchmark dataset plays a crucial role in the objective evaluation of segmentation methods, as they provide standardized and reproducible results that can be compared among different algorithms. By testing these algorithms on BDSD500, we can ensure that the performance of the algorithms is evaluated under different conditions.

Table 1 provides the main parameters of these algorithms. In all experiments, the population size is set to 20, and the maximum number of iterations is 100. For a fair comparison, all algorithms terminate when they reach the maximum of 2000 evaluations.

### 4.1. Experimental Analysis Based on Kapur Entropy

The proposed MGWO is compared with other algorithms based on the optimal objective function values and their variance, signal-to-noise ratio (PSNR), structural similarity index measure (SSIM), and feature similarity index (FSIM). To facilitate observation and analysis, the best results from the tables mentioned briefly below (averages of twenty runs) are highlighted in bold. We also use the Friedman and Wilcoxon rank-sum tests to validate the experimental results obtained.

Table 2 presents the best objective function values according to Kapur entropy. The solution quality improves as the threshold level increases. From the findings in Table 2, it can be seen that the proposed MGWO performs exceptionally well in 27 images, and it achieves 5, 8, 5, and 9 optimal solutions at threshold levels of 2, 3, 4, and 5, respectively. MGWO demonstrates outstanding segmentation ability at threshold levels 3 and 5. GWO and MWOA outperform their competitors in 6 and 7 images, respectively, while FOA performs the worst. The superiority of MGWO over GWO suggests that the strategy proposed in this paper is satisfactory for multi-level thresholding segmentation. The Kapur values of the algorithms in images 113044, 140075, 176019, 209070, and 288024 surpass those in the other images. The algorithms excel with an increased number of threshold levels. Images 8068 and 208001 significantly enhance performance when the threshold levels exceed 2.

By checking the experimental data through the Wilcoxon rank-sum test, it is found that GWO, MGWO, FOA, and MWOA perform well in 15, 34, 0, and 17 images, respectively. Their average ranks are 2.3, 1.55, 3.975, and 2.175, respectively. According to non-parametric statistical analysis, MGWO is better than the comparison algorithms. MGWO’s exceptional stability is demonstrated by its consistent performance across various images. The algorithm can generate reliable results in different images and has a strong ability to segment images.

Table 3 shows the average running time of the algorithms. Despite their short running times, the time taken increases with the number of threshold levels. The running time of FOA is obviously longer compared to the other algorithms. GWO ranks the lowest in terms of running time (1.7), while MGWO and MWOA share a similar rank (2.15). Due to its more intricate structure, MGWO takes longer than GWO. Nevertheless, its computation time remains manageable, and the average CPU time required by MGWO is less than 3%, which is completely acceptable for image segmentation.

**Figure 5:**
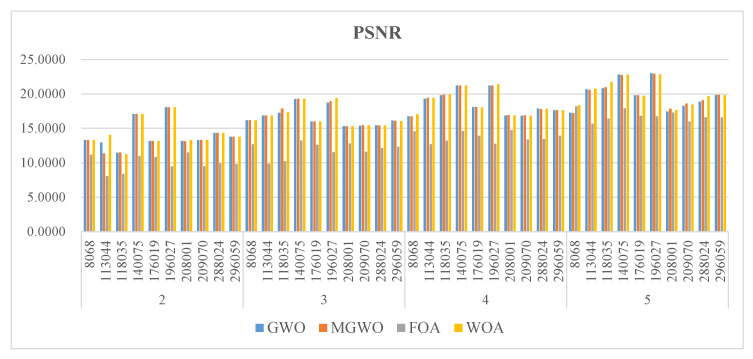
The PSNR of the algorithms.

**Figure 6:**
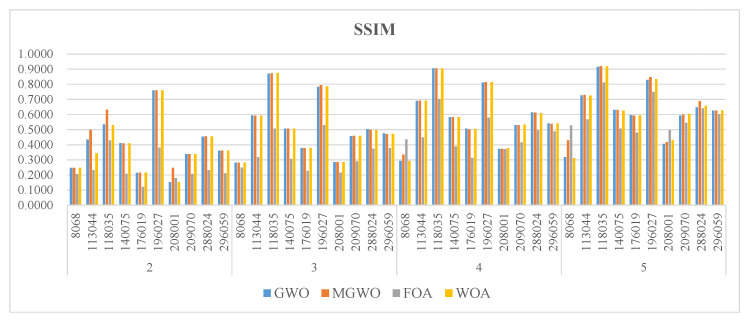
The SSIM of the algorithms.

**Figure 7:**
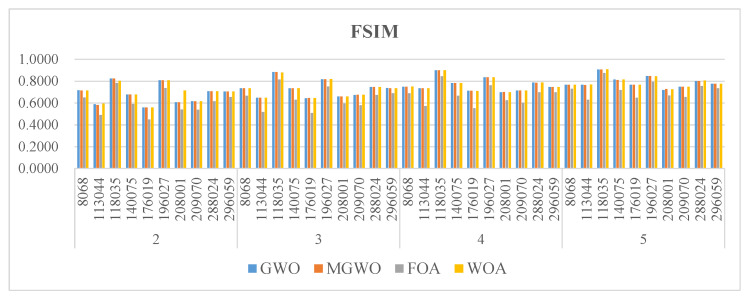
The FSIM of the algorithms.

Figure 5, Figure 6 and Figure 7 present the PSNR, SSIM, and FSIM values for the segmentation results based on Kapur entropy. PSNR is a classic metric for measuring image quality. It calculates the difference between segmented and original images. A higher PSNR value indicates that the segmented image is more similar to the original one. GWO, MGWO, FOA, and MWOA perform well in multiple images, achieving high PSNR values in 7, 19, 0, and 17 images. In particular, MGWO exhibits exceptional performance in several test images, especially at threshold levels of 2, 3, and 4, where it outperforms the other comparison algorithms. The excellent performance of MGWO can be attributed to its global search capability during the optimization process, and it effectively finds the optimal balance among multiple thresholds. MGWO successfully preserves the details and clarity of the images during segmentation.

SSIM measures the structural similarity between images and takes into account brightness, contrast, and structure. A higher SSIM value, closer to 1, means that the segmented image has higher structural similarity to the original image. MGWO achieves optimal solutions in 15 images, accounting for 37.5% of all test cases. In comparison, MWOA and GWO are effective in 11 images. Consequently, MGWO demonstrates higher SSIM values in most images, showcasing its superior structural similarity.

FSIM mainly measures the feature similarity between images, and it is often used for image quality assessment, especially in detail and texture comparisons. Although MGWO slightly lags behind MWOA in FSIM, it still performs excellently. It achieves the best FSIM values in 30 images, just 3 images behind MWOA. This result proves that MGWO is proficient at preserving the integrity of image features. Additionally, MGWO maintains high FSIM values in most images through its effective multi-threshold segmentation strategy.

### 4.2. Experimental Analysis Based on the Otsu Method

From Table 4, it is evident that MGWO based on the Otsu method has higher objective function values than GWO, MWOA, and FOA. It achieves the highest optimal objective function values in 28 out of 40 images, followed by MWOA, GWO, and FOA. The Otsu values of Images 8068, 118035, and 208001 are superior to those of the other images. The algorithms are more efficient with five threshold levels than with two, three, and four threshold levels. The Wilcoxon rank-sum test shows that MGWO achieves the best Otsu values on 34 images, followed by MWOA (20), GWO (8), and FOA. The average ranking of MGWO is superior to those of these algorithms. MGWO also demonstrates excellent performance in terms of variance. It exhibits robustness and minimal fluctuation across various images, and it is suitable for a wide range of applications.

Table 5 displays the running times of the algorithms. The WOA algorithm exhibits the fastest running time. GWO runs slightly faster than MGWO, while FOA is the slowest. Their running times are similar, and the maximum time difference between GWO and WOA is less than 2.6%. The algorithms, when used on the Otsu and Kapur values, show no significant time difference.

**Figure 8:**
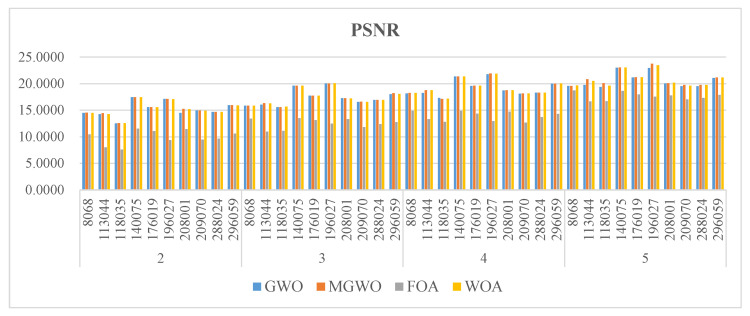
The PSNR of the algorithms.

**Figure 9:**
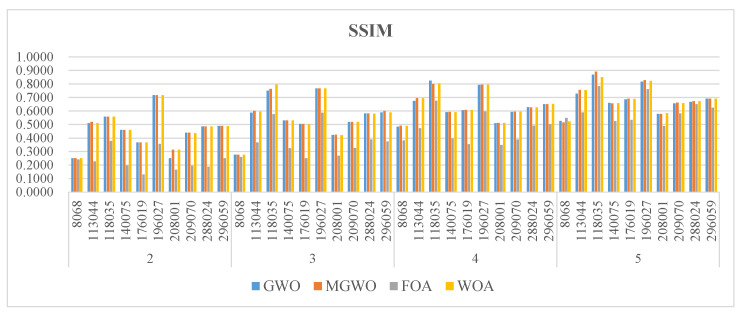
The SSIM of the algorithms.

**Figure 10:**
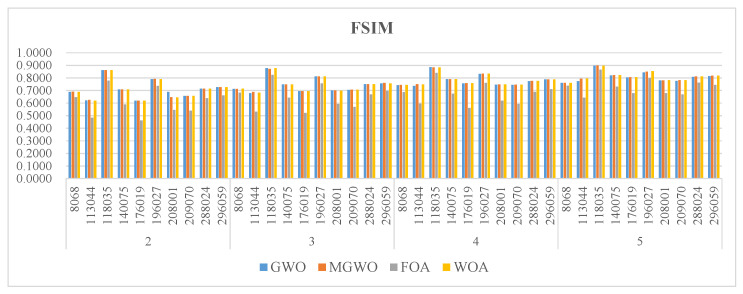
The FSIM of the algorithms.

Figure 8, Figure 9 and Figure 10 present the PSNR, SSIM, and FSIM values of the segmentation results based on the Otsu method. MGWO achieves the best PSNR values in 21 images, which is significantly better than other comparison algorithms. The Wilcoxon rank-sum test further validates MGWO’s outstanding performance because it obtains the optimal PSNR values in 32 images. This result indicates that MGWO is better at maintaining image quality across multiple images, reducing noise and unnecessary detail loss.

MGWO ranks among the top algorithms among all the compared algorithms, and it achieves the best values in 24 cases. The Wilcoxon rank-sum test results show that MGWO achieves the best SSIM values in 38 images and significantly outperforms MWOA, GWO, and FOA. This strong performance stems from MGWO’s optimization process, which combines global search with local adjustment strategies. This allows the algorithm to accurately identify optimal threshold points for segmentation and enhance the structural similarity of the images.

For FSIM, although MGWO performs slightly worse than MWOA, it has two fewer optimal solutions. It still shows excellent results. MGWO’s average rank is 1.725, better than MWOA’s 1.875. It remains more consistent compared to MWOA, particularly when facing changes in image content. MWOA’s results are more sensitive to differences in image features, which leads to more fluctuation. MGWO has stronger robustness when handling different types of images, and it is more effective in retaining key image features during multi-threshold segmentation.

### 4.3. Discussion

**Figure 11:**
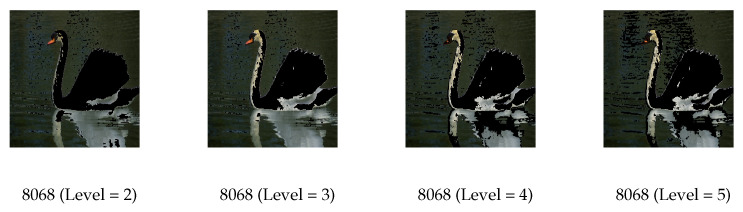

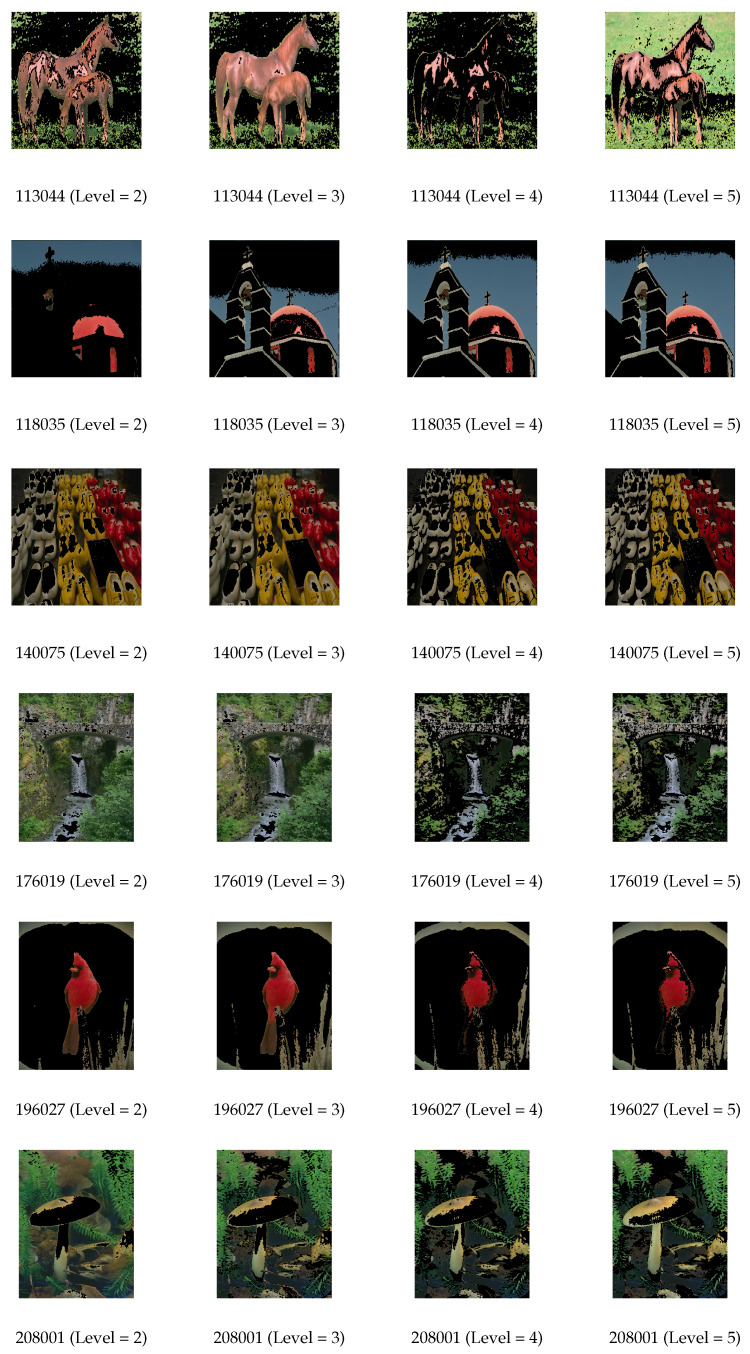

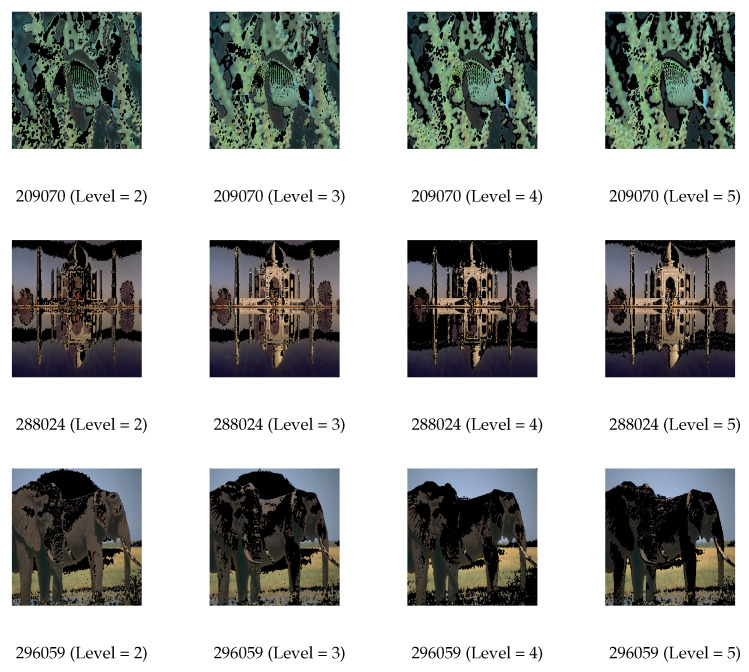
Results of thresholded images with different thresholding levels of MGWO based on Kapur entropy.

**Figure 12:**
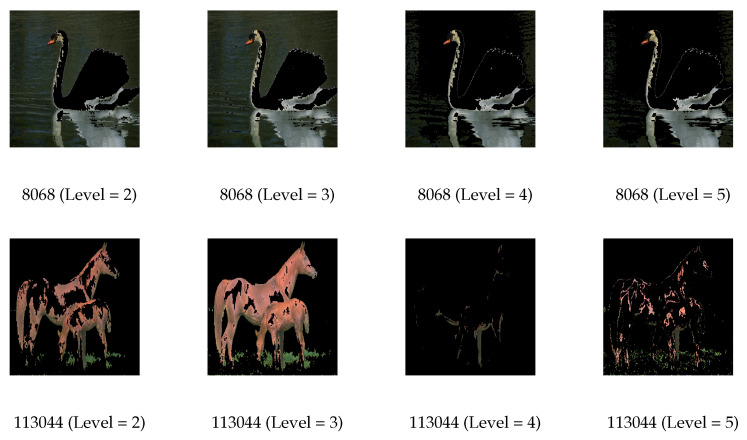

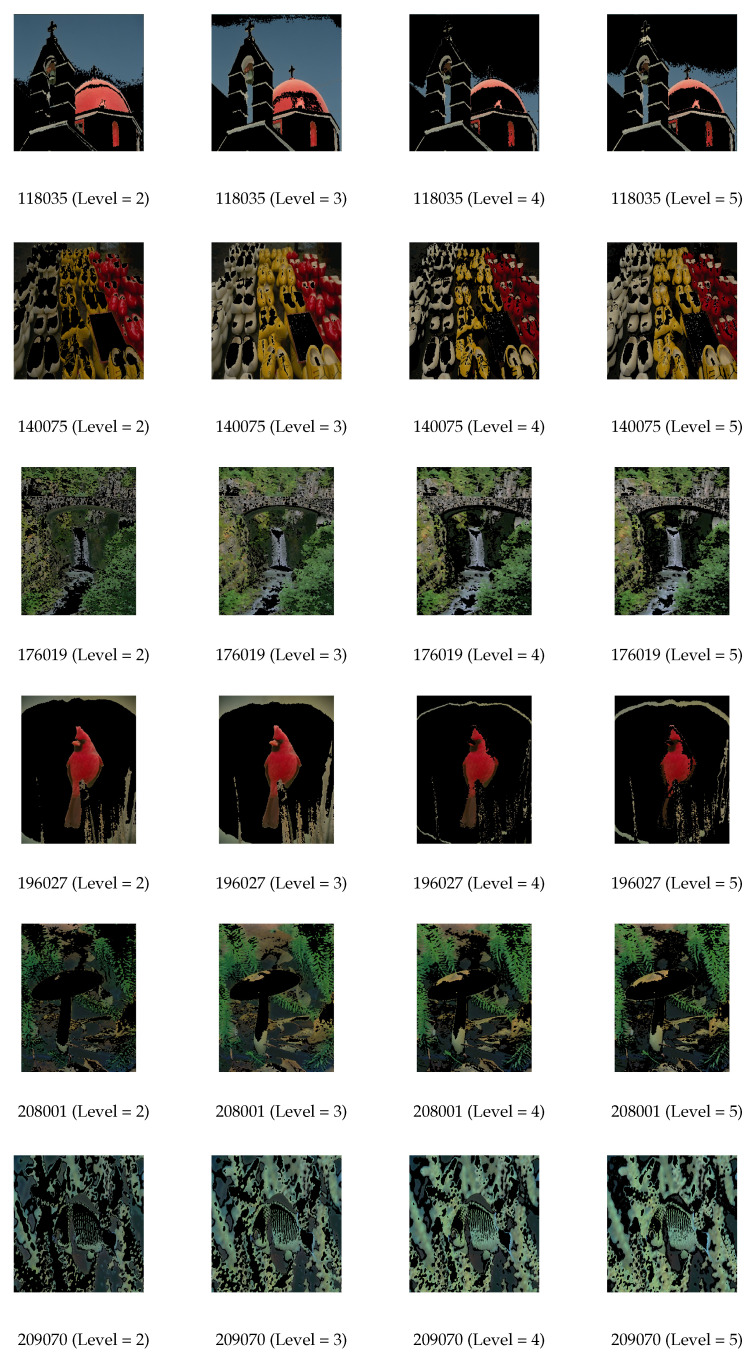

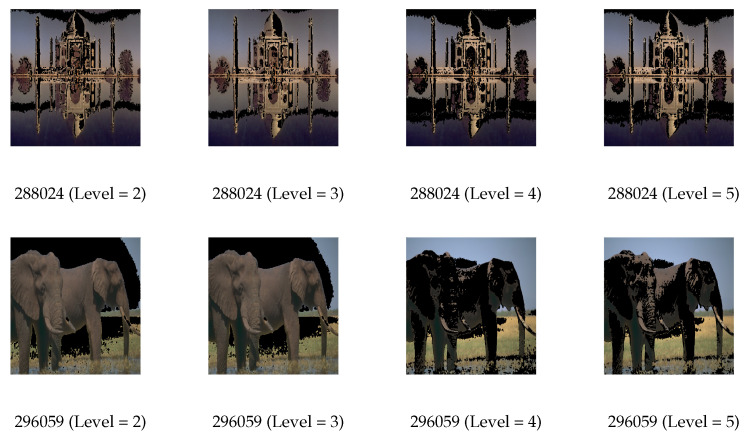
Results of thresholded images with different thresholding levels of MGWO based on the Otsu method.

Figure 11 and Figure 12 show the segmented images using Kapur and Otsu values under different threshold levels from the algorithms.

The segmented images become more vivid and detailed with an increase in the number of threshold levels. The images segmented by MGWO can be easily recognized visually but also exhibit weaknesses in certain areas. High threshold levels can provide more detailed segmentation; however, they may also cause unclear or overlapping region boundaries, especially when pixel intensities change gradually. MGWO may need help to accurately define these boundaries, which adversely affects the visual quality and interpretability of the segmented images. In the segmentation based on Kapur entropy, when the threshold level is 4 in Image 113044, the algorithm successfully separates the horses from the surrounding background but fails to capture the detailed features of the horses. The segmentation process typically divides an image into regions based on pixel intensity values, so it may have difficulty capturing the finer details of the horses when there is little variation in pixel intensity values within the horses’ region. On the other hand, MGWO handles various details at a threshold level of 5, due to its ability to balance global search and local exploitation, allowing it to capture finer variations in pixel intensity and preserve key image features at this threshold. In the Otsu method, MGWO does not perform well visually at either threshold level 4 or 5 in Image 113044. At these thresholds, the intensity values may be too close. MGWO is difficult to accurately segment the image, and it has poor visual performance. In Image 118035, MGWO does not completely distinguish the sky at threshold levels 4 and 5, instead mistakenly dividing it into two parts. MGWO misclassifies when the sky exhibits similar pixel values to nearby areas. In Image 296059, while MGWO captures the details of the animals well, it compromises the overall visual coherence of the elephant. In the detailed areas of the animal, the algorithm may overfit the segmentation by concentrating excessively on fine details, such as fur texture or contours, and compromise the overall structural coherence of the image.

## 5. Conclusions

This paper proposes a method that utilizes GWO to overcome the shortcomings of traditional multi-level thresholding color image segmentation algorithms. Otsu and Kapur are used as objective functions to find the optimal thresholds for multi-level segmentation. The MGWO algorithm is tested on ten color test images, considering four different threshold levels (2, 3, 4, and 5), and its performance is compared with the classical GWO algorithm and two state-of-the-art algorithms. The performance metrics include objective function values, variance, PSNR, SSIM, and FSIM. Our experiments reveal that MGWO performs exceptionally well on these metrics. Due to the randomness of meta-heuristic algorithms, we also analyze the obtained experimental data through the Friedman and Wilcoxon rank-sum tests, which further prove the performance of MGWO. Otsu and Kapur excel in multi-level segmentation tasks due to their established thresholding techniques, which efficiently reduce intra-class variance (Otsu) and increase entropy (Kapur). The effectiveness of these methods depends on their strong statistical foundations for determining optimal thresholds. Otsu works exceptionally well when there is a clear distinction between the foreground and background, whereas Kapur excels in handling more complex images with uneven pixel intensity distributions that contain unique patterns or textures. Otsu tends to perform poorly when an image contains multiple objects with similar intensity levels. Since Otsu assumes a bimodal distribution, it may fail to capture complex scenes characterized by overlapping or multimodal histograms. Furthermore, it has difficulty processing low-contrast images. On the other hand, Kapur can be sensitive to regions with subtle intensity variations. Additionally, Kapur can sometimes lead to over-segmentation and generate unnecessary divisions or blurry boundaries.

In the future, we intend to introduce additional evaluation criteria to validate the robustness of MGWO. We also plan to apply it to complex image processing applications, such as dam crack detection. Since GWO is a popular meta-heuristic algorithm, there is potential to explore its recent advancements and implement them in the multi-level thresholding segmentation of color images to improve recognition results.

## Figures and Tables

**Table 1:** The key parameters of the compared algorithms.

Algorithm	Key Parameters
GWO & MGWO	a = 2 (Equation (14)).
FOA	area_limit (limit of trees in the forest) = 200;
	Life_time (age limit to be part of the candidate list) = 15;
	Transfer_rate (percentage of the trees in the candidate list that are going to global seed) = 10.
WOA	a = 2; a2 = −1; b = 1 (parameters to control update position).

**Table 2:** The fitness values of the algorithms.

Level	Image	GWO		MGWO		FOA		MWOA	
		Value	Variance	Value	Variance	Value	Variance	Value	Variance
2	8068	1.1902 × $10^{1}$	8.5419 × $10^{-10}$	**1.1902 × $10^{1}$**	7.6804 × $10^{-11}$	1.1870 × $10^{1}$	9.8410 × $10^{-4}$	1.1902 × $10^{1}$	2.9956 × $10^{-9}$
	113044	1.2121 × $10^{1}$	7.7357 × $10^{-4}$	1.2100 × $10^{1}$	1.3179 × $10^{-5}$	1.2039 × $10^{1}$	1.1184 × $10^{-2}$	**1.2153 × $10^{1}$**	1.4094 × $10^{-4}$
	118035	1.0844 × $10^{1}$	2.3426 × $10^{-5}$	1.0830 × $10^{1}$	1.2965 × $10^{-5}$	1.0716 × $10^{1}$	9.7160 × $10^{-3}$	**1.0845 × $10^{1}$**	2.5778 × $10^{-7}$
	140075	1.2350 × $10^{1}$	1.3491 × $10^{-7}$	**1.2350 × $10^{1}$**	1.0143 × $10^{-8}$	1.2288 × $10^{1}$	1.0685 × $10^{-2}$	1.2350 × $10^{1}$	5.5595 × $10^{-9}$
	176019	1.2696 × $10^{1}$	1.6421 × $10^{-7}$	**1.2696 × $10^{1}$**	4.0287 × $10^{-8}$	1.2658 × $10^{1}$	1.4973 × $10^{-3}$	1.2696 × $10^{1}$	1.0097 × $10^{-7}$
	196027	1.1768 × $10^{1}$	4.6764 × $10^{-8}$	1.1768 × $10^{1}$	3.1959 × $10^{-8}$	1.1533 × $10^{1}$	1.4548 × $10^{-1}$	**1.1768 × $10^{1}$**	1.0121 × $10^{-8}$
	208001	**1.2446 × $10^{1}$**	1.5307 × $10^{-9}$	1.1902 × $10^{1}$	1.2827 × $10^{-10}$	1.2401 × $10^{1}$	2.2423 × $10^{-3}$	1.2446 × $10^{1}$	4.6799 × $10^{-7}$
	209070	1.2563 × $10^{1}$	2.9000 × $10^{-7}$	**1.2564 × $10^{1}$**	9.8704 × $10^{-9}$	1.2540 × $10^{1}$	6.3441 × $10^{-4}$	1.2563 × $10^{1}$	3.1640 × $10^{-8}$
	288024	1.2606 × $10^{1}$	8.2327 × $10^{-8}$	1.2606 × $10^{1}$	4.4667 × $10^{-9}$	1.2565 × $10^{1}$	9.2084 × $10^{-4}$	**1.2606 × $10^{1}$**	3.5134 × $10^{-9}$
	296059	1.2198 × $10^{1}$	6.9453 × $10^{-7}$	**1.2198 × $10^{1}$**	4.1639 × $10^{-10}$	1.2109 × $10^{1}$	1.0636 × $10^{-2}$	1.2198 × $10^{1}$	3.1262 × $10^{-8}$
3	8068	1.5274 × $10^{1}$	2.1798 × $10^{-7}$	**1.5274 × $10^{1}$**	9.0891 × $10^{-8}$	1.5224 × $10^{1}$	8.4500 × $10^{-3}$	1.5273 × $10^{1}$	2.7010 × $10^{-7}$
	113044	1.5327 × $10^{1}$	6.3871 × $10^{-6}$	**1.5329 × $10^{1}$**	2.3435 × $10^{-6}$	1.5154 × $10^{1}$	3.9407 × $10^{-3}$	1.5328 × $10^{1}$	3.2677 × $10^{-6}$
	118035	1.4276 × $10^{1}$	4.4832 × $10^{-4}$	1.4247 × $10^{1}$	5.1335 × $10^{-3}$	1.4149 × $10^{1}$	1.6903 × $10^{-2}$	**1.4302 × $10^{1}$**	1.8138 × $10^{-4}$
	140075	1.5433 × $10^{1}$	5.6939 × $10^{-7}$	**1.5433 × $10^{1}$**	2.0135 × $10^{-8}$	1.5331 × $10^{1}$	4.5250 × $10^{-3}$	1.5433 × $10^{1}$	5.6873 × $10^{-8}$
	176019	1.5856 × $10^{1}$	1.3274 × $10^{-7}$	**1.5856 × $10^{1}$**	2.0694 × $10^{-7}$	1.5808 × $10^{1}$	4.3773 × $10^{-3}$	1.5855 × $10^{1}$	4.3987 × $10^{-7}$
	196027	**1.4668 × $10^{1}$**	1.6506 × $10^{-4}$	1.4632 × $10^{1}$	1.2371 × $10^{-4}$	1.4312 × $10^{1}$	7.3531 × $10^{-2}$	1.4655 × $10^{1}$	5.4111 × $10^{-4}$
	208001	1.5478 × $10^{1}$	1.5551 × $10^{-7}$	**1.5478 × $10^{1}$**	5.2621 × $10^{-8}$	1.5326 × $10^{1}$	4.1266 × $10^{-2}$	1.5478 × $10^{1}$	2.2421 × $10^{-7}$
	209070	1.5685 × $10^{1}$	1.2024 × $10^{-6}$	**1.5686 × $10^{1}$**	3.6814 × $10^{-7}$	1.5629 × $10^{1}$	1.8334 × $10^{-3}$	1.5685 × $10^{1}$	3.8213 × $10^{-7}$
	288024	1.5666 × $10^{1}$	4.4438 × $10^{-6}$	**1.5668 × $10^{1}$**	1.4162 × $10^{-7}$	1.5572 × $10^{1}$	1.5380 × $10^{-3}$	1.5666 × $10^{1}$	2.6642 × $10^{-6}$
	296059	1.5176 × $10^{1}$	2.1353 × $10^{-5}$	**1.5179 × $10^{1}$**	2.6285 × $10^{-6}$	1.5073 × $10^{1}$	1.5682 × $10^{-3}$	1.5179 × $10^{1}$	1.9770 × $10^{-6}$
4	8068	**1.8342 × $10^{1}$**	9.2740 × $10^{-6}$	1.8282 × $10^{1}$	9.6670 × $10^{-3}$	1.8027 × $10^{1}$	5.5451 × $10^{-2}$	1.8340 × $10^{1}$	1.3507 × $10^{-5}$
	113044	1.8157 × $10^{1}$	8.8248 × $10^{-4}$	**1.8166 × $10^{1}$**	3.6804 × $10^{-6}$	1.7510 × $10^{1}$	1.9608 × $10^{-1}$	1.8161 × $10^{1}$	1.5968 × $10^{-5}$
	118035	1.7540 × $10^{1}$	3.2793 × $10^{-4}$	1.7543 × $10^{1}$	2.3837 × $10^{-3}$	1.6818 × $10^{1}$	1.3857 × $10^{-1}$	**1.7543 × $10^{1}$**	4.9621 × $10^{-5}$
	140075	1.8262 × $10^{1}$	3.6488 × $10^{-7}$	**1.8262 × $10^{1}$**	1.8918 × $10^{-7}$	1.7482 × $10^{1}$	2.6341 × $10^{-1}$	1.8261 × $10^{1}$	1.4499 × $10^{-6}$
	176019	**1.8794 × $10^{1}$**	1.1416 × $10^{-6}$	1.8794 × $10^{1}$	3.4774 × $10^{-6}$	1.8457 × $10^{1}$	6.2378 × $10^{-2}$	1.8792 × $10^{1}$	2.6159 × $10^{-6}$
	196027	1.7373 × $10^{1}$	1.2662 × $10^{-4}$	1.7370 × $10^{1}$	1.0765 × $10^{-3}$	1.6005 × $10^{1}$	3.0660 × $10^{-1}$	**1.7381 × $10^{1}$**	3.4051 × $10^{-5}$
	208001	1.8302 × $10^{1}$	1.4680 × $10^{-6}$	**1.8303 × $10^{1}$**	1.8772 × $10^{-7}$	1.7942 × $10^{1}$	9.9034 × $10^{-2}$	1.8300 × $10^{1}$	2.9631 × $10^{-6}$
	209070	**1.8562 × $10^{1}$**	1.6392 × $10^{-6}$	1.8562 × $10^{1}$	1.2052 × $10^{-6}$	1.8122 × $10^{1}$	1.5156 × $10^{-1}$	1.8560 × $10^{1}$	2.9888 × $10^{-6}$
	288024	1.8532 × $10^{1}$	7.7414 × $10^{-6}$	**1.8534 × $10^{1}$**	3.4938 × $10^{-6}$	1.8075 × $10^{1}$	6.9957 × $10^{-2}$	1.8530 × $10^{1}$	1.9334 × $10^{-5}$
	296059	1.7994 × $10^{1}$	1.8631 × $10^{-4}$	**1.8009 × $10^{1}$**	9.5785 × $10^{-6}$	1.7494 × $10^{1}$	2.2678 × $10^{-1}$	1.8003 × $10^{1}$	2.1074 × $10^{-5}$
5	8068	2.0976 × $10^{1}$	8.5506 × $10^{-3}$	**2.1085 × $10^{1}$**	4.6980 × $10^{-4}$	2.0530 × $10^{1}$	2.8209 × $10^{-2}$	2.1080 × $10^{1}$	5.2297 × $10^{-5}$
	113044	2.0735 × $10^{1}$	1.8430 × $10^{-4}$	**2.0738 × $10^{1}$**	2.2087 × $10^{-4}$	1.9440 × $10^{1}$	2.9525 × $10^{-1}$	2.0722 × $10^{1}$	2.3028 × $10^{-4}$
	118035	2.0154 × $10^{1}$	2.6834 × $10^{-3}$	**2.0188 × $10^{1}$**	1.6981 × $10^{-3}$	1.9159 × $10^{1}$	6.7358 × $10^{-2}$	2.0171 × $10^{1}$	8.8101 × $10^{-4}$
	140075	2.0863 × $10^{1}$	6.6966 × $10^{-6}$	**2.0864 × $10^{1}$**	5.6506 × $10^{-7}$	1.9480 × $10^{1}$	2.2544 × $10^{-1}$	2.0856 × $10^{1}$	2.8546 × $10^{-5}$
	176019	2.1540 × $10^{1}$	6.0726 × $10^{-6}$	**2.1542 × $10^{1}$**	7.6179 × $10^{-7}$	2.1008 × $10^{1}$	6.3971 × $10^{-2}$	2.1535 × $10^{1}$	1.3808 × $10^{-5}$
	196027	1.9776 × $10^{1}$	1.3590 × $10^{-3}$	**1.9816 × $10^{1}$**	3.1380 × $10^{-4}$	1.7445 × $10^{1}$	3.1206 × $10^{-1}$	1.9801 × $10^{1}$	4.5807 × $10^{-4}$
	208001	2.0919 × $10^{1}$	4.7231 × $10^{-5}$	**2.0926 × $10^{1}$**	1.8102 × $10^{-6}$	1.9907 × $10^{1}$	1.7524 × $10^{-1}$	2.0907 × $10^{1}$	1.6447 × $10^{-4}$
	209070	2.1230 × $10^{1}$	2.9622 × $10^{-5}$	**2.1234 × $10^{1}$**	2.2795 × $10^{-6}$	2.0418 × $10^{1}$	1.5892 × $10^{-1}$	2.1217 × $10^{1}$	5.7495 × $10^{-5}$
	288024	2.1192 × $10^{1}$	2.5612 × $10^{-3}$	**2.1242 × $10^{1}$**	4.4796 × $10^{-5}$	2.0601 × $10^{1}$	2.7745 × $10^{-2}$	2.1206 × $10^{1}$	2.0630 × $10^{-3}$
	296059	**2.0600 × $10^{1}$**	1.0276 × $10^{-5}$	2.0585 × $10^{1}$	2.4028 × $10^{-4}$	1.9147 × $10^{1}$	1.9505 × $10^{-1}$	2.0589 × $10^{1}$	5.7304 × $10^{-5}$
	>/=/<	6/9/25		27/7/6		0/0/40		7/10/23	
	Rank	2.3		1.55		3.975		2.175	

**Table 3:** The average running time of the algorithms (seconds).

Level	Image	GWO	MGWO	FOA	MWOA
2	8068	2.5348 × $10^{0}$	2.5382 × $10^{0}$	3.4200 × $10^{0}$	2.5263 × $10^{0}$
	113044	2.5143 × $10^{0}$	2.5259 × $10^{0}$	3.4328 × $10^{0}$	2.4717 × $10^{0}$
	118035	2.5345 × $10^{0}$	2.5301 × $10^{0}$	3.4685 × $10^{0}$	2.4898 × $10^{0}$
	140075	2.5350 × $10^{0}$	2.5463 × $10^{0}$	3.4592 × $10^{0}$	2.5923 × $10^{0}$
	176019	2.5345 × $10^{0}$	2.5388 × $10^{0}$	3.5196 × $10^{0}$	2.5383 × $10^{0}$
	196027	2.4593 × $10^{0}$	2.4539 × $10^{0}$	3.5104 × $10^{0}$	2.4501 × $10^{0}$
	208001	2.5283 × $10^{0}$	2.5377 × $10^{0}$	3.5156 × $10^{0}$	2.5591 × $10^{0}$
	209070	2.5282 × $10^{0}$	2.5140 × $10^{0}$	3.5788 × $10^{0}$	2.5521 × $10^{0}$
	288024	2.5025 × $10^{0}$	2.5114 × $10^{0}$	3.5579 × $10^{0}$	2.5341 × $10^{0}$
	296059	2.5120 × $10^{0}$	2.5187 × $10^{0}$	3.5562 × $10^{0}$	2.5351 × $10^{0}$
3	8068	2.8955 × $10^{0}$	2.9130 × $10^{0}$	3.8698 × $10^{0}$	2.8439 × $10^{0}$
	113044	2.8000 × $10^{0}$	2.8120 × $10^{0}$	3.7716 × $10^{0}$	2.7619 × $10^{0}$
	118035	2.8276 × $10^{0}$	2.8155 × $10^{0}$	3.7910 × $10^{0}$	2.7790 × $10^{0}$
	140075	2.8783 × $10^{0}$	2.8889 × $10^{0}$	3.8775 × $10^{0}$	2.9603 × $10^{0}$
	176019	2.8830 × $10^{0}$	2.8968 × $10^{0}$	3.9531 × $10^{0}$	2.8768 × $10^{0}$
	196027	2.7268 × $10^{0}$	2.7404 × $10^{0}$	3.8260 × $10^{0}$	2.7563 × $10^{0}$
	208001	2.8871 × $10^{0}$	2.8941 × $10^{0}$	3.8968 × $10^{0}$	2.9099 × $10^{0}$
	209070	2.8663 × $10^{0}$	2.8541 × $10^{0}$	3.9595 × $10^{0}$	2.8975 × $10^{0}$
	288024	2.8397 × $10^{0}$	2.8279 × $10^{0}$	3.9290 × $10^{0}$	2.8445 × $10^{0}$
	296059	2.8283 × $10^{0}$	2.8343 × $10^{0}$	3.9252 × $10^{0}$	2.8549 × $10^{0}$
4	8068	3.4369 × $10^{0}$	3.4557 × $10^{0}$	4.3947 × $10^{0}$	3.3829 × $10^{0}$
	113044	3.3124 × $10^{0}$	3.3248 × $10^{0}$	4.2480 × $10^{0}$	3.2801 × $10^{0}$
	118035	3.3064 × $10^{0}$	3.3130 × $10^{0}$	4.2654 × $10^{0}$	3.2662 × $10^{0}$
	140075	3.3994 × $10^{0}$	3.4010 × $10^{0}$	4.4154 × $10^{0}$	3.6151 × $10^{0}$
	176019	3.4107 × $10^{0}$	3.4164 × $10^{0}$	4.4972 × $10^{0}$	3.3982 × $10^{0}$
	196027	3.2195 × $10^{0}$	3.2093 × $10^{0}$	4.2661 × $10^{0}$	3.2080 × $10^{0}$
	208001	3.4092 × $10^{0}$	3.4148 × $10^{0}$	4.4104 × $10^{0}$	3.8744 × $10^{0}$
	209070	3.4592 × $10^{0}$	3.4773 × $10^{0}$	4.4953 × $10^{0}$	3.4876 × $10^{0}$
	288024	3.3324 × $10^{0}$	3.3423 × $10^{0}$	4.4625 × $10^{0}$	3.3513 × $10^{0}$
	296059	3.3648 × $10^{0}$	3.3581 × $10^{0}$	4.4335 × $10^{0}$	3.4058 × $10^{0}$
5	8068	4.0045 × $10^{0}$	4.0751 × $10^{0}$	4.9174 × $10^{0}$	4.0295 × $10^{0}$
	113044	3.8784 × $10^{0}$	3.8006 × $10^{0}$	4.6178 × $10^{0}$	3.7766 × $10^{0}$
	118035	3.8350 × $10^{0}$	3.8109 × $10^{0}$	4.7504 × $10^{0}$	3.7850 × $10^{0}$
	140075	3.9455 × $10^{0}$	3.9303 × $10^{0}$	4.8520 × $10^{0}$	4.0127 × $10^{0}$
	176019	4.0878 × $10^{0}$	4.0986 × $10^{0}$	4.8714 × $10^{0}$	4.0538 × $10^{0}$
	196027	3.7872 × $10^{0}$	3.7797 × $10^{0}$	4.7204 × $10^{0}$	3.8031 × $10^{0}$
	208001	4.1469 × $10^{0}$	4.1753 × $10^{0}$	4.8827 × $10^{0}$	4.5643 × $10^{0}$
	209070	3.9760 × $10^{0}$	4.0034 × $10^{0}$	4.9757 × $10^{0}$	4.0339 × $10^{0}$
	288024	3.9730 × $10^{0}$	3.9829 × $10^{0}$	4.8832 × $10^{0}$	3.9840 × $10^{0}$
	296059	3.8812 × $10^{0}$	3.9036 × $10^{0}$	4.8788 × $10^{0}$	3.9630 × $10^{0}$

**Table 4:** The fitness values of the algorithms.

Level	Image	GWO		MGWO		FOA		MWOA	
		Value	Variance	Value	Variance	Value	Variance	Value	Variance
2	8068	3.7513 × $10^{3}$	4.2126 × $10^{-5}$	**3.7513 × $10^{3}$**	5.9854 × $10^{-6}$	3.7404 × $10^{3}$	4.2340 × $10^{2}$	3.7513 × $10^{3}$	5.9854 × $10^{-6}$
	113044	**1.1662 × $10^{3}$**	2.7576 × $10^{-4}$	1.1514 × $10^{3}$	1.0889 × $10^{3}$	1.1419 × $10^{3}$	6.4290 × $10^{2}$	1.1537 × $10^{3}$	1.0477 × $10^{3}$
	118035	3.0857 × $10^{3}$	1.8415 × $10^{-5}$	**3.0857 × $10^{3}$**	3.2344 × $10^{-6}$	3.0706 × $10^{3}$	3.2083 × $10^{2}$	3.0833 × $10^{3}$	1.2223 × $10^{2}$
	140075	2.7493 × $10^{3}$	1.9019 × $10^{-4}$	2.7493 × $10^{3}$	7.7258 × $10^{-4}$	2.7105 × $10^{3}$	1.3699 × $10^{3}$	**2.7493 × $10^{3}$**	3.5547 × $10^{-6}$
	176019	1.2287 × $10^{3}$	2.6044 × $10^{-4}$	**1.2287 × $10^{3}$**	0.0000 × $10^{0}$	1.2073 × $10^{3}$	2.1838 × $10^{2}$	1.2287 × $10^{3}$	0.0000 × $10^{0}$
	196027	1.2008 × $10^{3}$	2.6979 × $10^{-4}$	1.1979 × $10^{3}$	1.7265 × $10^{2}$	1.1744 × $10^{3}$	6.5826 × $10^{2}$	**1.2008 × $10^{3}$**	6.1211 × $10^{-5}$
	208001	1.5725 × $10^{3}$	6.9609 × $10^{-5}$	1.5725 × $10^{3}$	0.0000 × $10^{0}$	1.5343 × $10^{3}$	1.1406 × $10^{3}$	**3.7513 × $10^{3}$**	1.3556 × $10^{-5}$
	209070	**1.5942 × $10^{3}$**	1.1088 × $10^{-4}$	1.5877 × $10^{3}$	8.4468 × $10^{2}$	1.5537 × $10^{3}$	1.8792 × $10^{3}$	1.5942 × $10^{3}$	8.6521 × $10^{-7}$
	288024	1.6975 × $10^{3}$	2.0477 × $10^{-4}$	**1.6975 × $10^{3}$**	2.1768 × $10^{-25}$	1.6806 × $10^{3}$	6.0779 × $10^{2}$	1.6975 × $10^{3}$	9.3242 × $10^{-5}$
	296059	1.7013 × $10^{3}$	1.6275 × $10^{-4}$	1.7013 × $10^{3}$	1.5119 × $10^{-4}$	1.6585 × $10^{3}$	2.1447 × $10^{3}$	**1.7013 × $10^{3}$**	6.1282 × $10^{-5}$
3	8068	3.8286 × $10^{3}$	6.0611 × $10^{-4}$	**3.8286 × $10^{3}$**	1.7918 × $10^{-5}$	3.8260 × $10^{3}$	2.4600 × $10^{0}$	3.8286 × $10^{3}$	2.3636 × $10^{-5}$
	113044	1.2506 × $10^{3}$	5.5267 × $10^{-3}$	1.2426 × $10^{3}$	1.7243 × $10^{2}$	1.2145 × $10^{3}$	2.2393 × $10^{2}$	**1.2377 × $10^{3}$**	2.9128 × $10^{2}$
	118035	**3.1305 × $10^{3}$**	2.1187 × $10^{-1}$	3.1286 × $10^{3}$	5.8529 × $10^{0}$	3.1205 × $10^{3}$	1.2130 × $10^{2}$	3.1273 × $10^{3}$	3.9913 × $10^{1}$
	140075	2.8992 × $10^{3}$	1.5157 × $10^{-3}$	**2.8993 × $10^{3}$**	3.3348 × $10^{-4}$	2.8365 × $10^{3}$	2.4574 × $10^{3}$	2.8993 × $10^{3}$	1.4402 × $10^{-4}$
	176019	1.3525 × $10^{3}$	2.4362 × $10^{-3}$	1.3507 × $10^{3}$	6.8789 × $10^{1}$	1.3243 × $10^{3}$	5.2852 × $10^{2}$	**1.3525 × $10^{3}$**	3.8564 × $10^{-5}$
	196027	1.2941 × $10^{3}$	6.1103 × $10^{-3}$	**1.2941 × $10^{3}$**	7.4134 × $10^{-4}$	1.2166 × $10^{3}$	2.2517 × $10^{3}$	1.2942 × $10^{3}$	6.7370 × $10^{-4}$
	208001	1.6870 × $10^{3}$	2.2851 × $10^{-3}$	**1.6870 × $10^{3}$**	5.4420 × $10^{-26}$	1.6585 × $10^{3}$	6.4638 × $10^{2}$	1.6870 × $10^{3}$	3.2663 × $10^{-4}$
	209070	1.7069 × $10^{3}$	2.3414 × $10^{-3}$	**1.7070 × $10^{3}$**	8.6371 × $10^{-6}$	1.6795 × $10^{3}$	3.4063 × $10^{2}$	1.7051 × $10^{3}$	6.6984 × $10^{1}$
	288024	1.8675 × $10^{3}$	7.6363 × $10^{-4}$	**1.8675 × $10^{3}$**	2.5607 × $10^{-4}$	1.8429 × $10^{3}$	3.6138 × $10^{2}$	1.8675 × $10^{3}$	1.7371 × $10^{-4}$
	296059	**1.8018 × $10^{3}$**	1.2265 × $10^{-3}$	1.8015 × $10^{3}$	4.9475 × $10^{-1}$	1.7534 × $10^{3}$	1.4136 × $10^{3}$	1.8003 × $10^{3}$	3.1988 × $10^{1}$
4	8068	3.8811 × $10^{3}$	7.2838 × $10^{-3}$	**3.8812 × $10^{3}$**	7.6560 × $10^{-5}$	3.8495 × $10^{3}$	1.8577 × $10^{2}$	3.8797 × $10^{3}$	2.1848 × $10^{1}$
	113044	1.2936 × $10^{3}$	5.6506 × $10^{-1}$	**1.2921 × $10^{3}$**	3.1971 × $10^{1}$	1.2333 × $10^{3}$	5.7124 × $10^{2}$	1.2832 × $10^{3}$	1.2498 × $10^{2}$
	118035	3.1581 × $10^{3}$	7.6005 × $10^{-2}$	**3.1579 × $10^{3}$**	9.1017 × $10^{-1}$	3.1392 × $10^{3}$	1.1621 × $10^{2}$	**3.1560 × $10^{3}$**	1.5965 × $10^{1}$
	140075	2.9667 × $10^{3}$	5.4818 × $10^{-2}$	**2.9669 × $10^{3}$**	3.2778 × $10^{-4}$	2.8818 × $10^{3}$	9.6639 × $10^{2}$	2.9669 × $10^{3}$	9.2454 × $10^{-4}$
	176019	1.4138 × $10^{3}$	1.9875 × $10^{-2}$	1.4140 × $10^{3}$	2.1885 × $10^{-6}$	1.3610 × $10^{3}$	7.4662 × $10^{2}$	1.4130 × $10^{3}$	2.1116 × $10^{1}$
	196027	1.3308 × $10^{3}$	1.5310 × $10^{-1}$	1.3305 × $10^{3}$	8.5334 × $10^{0}$	1.2496 × $10^{3}$	1.1096 × $10^{3}$	**1.3280 × $10^{3}$**	3.6646 × $10^{1}$
	208001	1.7460 × $10^{3}$	1.1270 × $10^{-2}$	**1.7461 × $10^{3}$**	2.5713 × $10^{-5}$	1.6900 × $10^{3}$	1.2028 × $10^{3}$	1.7456 × $10^{3}$	5.2104 × $10^{0}$
	209070	1.7678 × $10^{3}$	8.7322 × $10^{-3}$	**1.7680 × $10^{3}$**	9.1547 × $10^{-5}$	1.7183 × $10^{3}$	6.4965 × $10^{2}$	1.7651 × $10^{3}$	5.0130 × $10^{1}$
	288024	1.9224 × $10^{3}$	1.0740 × $10^{-2}$	**1.9226 × $10^{3}$**	6.0528 × $10^{-5}$	1.8675 × $10^{3}$	6.4144 × $10^{2}$	1.9207 × $10^{3}$	3.3265 × $10^{1}$
	296059	1.8627 × $10^{3}$	9.0410 × $10^{-3}$	**1.8628 × $10^{3}$**	3.4808 × $10^{-4}$	1.7971 × $10^{3}$	1.2264 × $10^{3}$	1.8613 × $10^{3}$	2.3305 × $10^{1}$
5	8068	3.9041 × $10^{3}$	9.7653 × $10^{-2}$	**3.9044 × $10^{3}$**	4.4262 × $10^{-5}$	3.8837 × $10^{3}$	9.1217 × $10^{1}$	3.9033 × $10^{3}$	5.1813 × $10^{0}$
	113044	1.3120 × $10^{3}$	2.3389 × $10^{1}$	**1.3166 × $10^{3}$**	1.3106 × $10^{1}$	1.2584 × $10^{3}$	1.5393 × $10^{2}$	1.3050 × $10^{3}$	6.0236 × $10^{1}$
	118035	3.1745 × $10^{3}$	2.1484 × $10^{-1}$	**3.1752 × $10^{3}$**	4.1850 × $10^{-1}$	3.1532 × $10^{3}$	2.0832 × $10^{1}$	3.1716 × $10^{3}$	1.0546 × $10^{1}$
	140075	3.0055 × $10^{3}$	1.3296 × $10^{-1}$	**3.0060 × $10^{3}$**	1.9376 × $10^{-3}$	2.9367 × $10^{3}$	2.0392 × $10^{2}$	3.0043 × $10^{3}$	1.7942 × $10^{1}$
	176019	1.4473 × $10^{3}$	5.5752 × $10^{-2}$	**1.4477 × $10^{3}$**	2.0612 × $10^{-5}$	1.3862 × $10^{3}$	2.9378 × $10^{2}$	1.4465 × $10^{3}$	1.2777 × $10^{1}$
	196027	1.3476 × $10^{3}$	2.0516 × $10^{0}$	**1.3504 × $10^{3}$**	1.0507 × $10^{-1}$	1.2874 × $10^{3}$	1.2368 × $10^{2}$	1.3410 × $10^{3}$	3.6864 × $10^{1}$
	208001	1.7747 × $10^{3}$	1.3073 × $10^{-1}$	**1.7752 × $10^{3}$**	5.2632 × $10^{-6}$	1.7292 × $10^{3}$	2.7469 × $10^{2}$	1.7711 × $10^{3}$	3.3760 × $10^{1}$
	209070	1.8009 × $10^{3}$	2.4700 × $10^{-1}$	**1.8018 × $10^{3}$**	1.4600 × $10^{-5}$	1.7378 × $10^{3}$	2.3626 × $10^{2}$	1.7930 × $10^{3}$	1.0632 × $10^{2}$
	288024	1.9588 × $10^{3}$	1.9516 × $10^{-1}$	**1.9594 × $10^{3}$**	1.5040 × $10^{-5}$	1.9011 × $10^{3}$	3.4154 × $10^{2}$	1.9546 × $10^{3}$	6.5146 × $10^{1}$
	296059	1.8893 × $10^{3}$	1.2565 × $10^{-1}$	**1.8899 × $10^{3}$**	1.2159 × $10^{-4}$	1.8175 × $10^{3}$	3.2412 × $10^{2}$	1.8851 × $10^{3}$	4.6062 × $10^{1}$
	>/=/<	4/4/32		28/6/6		0/0/40		8/12/20	
	Rank	2.30		1.625		4		2.075	

**Table 5:** The average running time of the algorithms (seconds).

Level	Image	GWO	MGWO	FOA	MWOA
2	8068	2.5501 × $10^{0}$	2.5150 × $10^{0}$	3.4924 × $10^{0}$	2.5048 × $10^{0}$
	113044	2.4913 × $10^{0}$	2.5302 × $10^{0}$	3.4126 × $10^{0}$	2.4566 × $10^{0}$
	118035	2.4775 × $10^{0}$	2.4894 × $10^{0}$	3.4401 × $10^{0}$	2.4278 × $10^{0}$
	140075	2.5015 × $10^{0}$	2.5057 × $10^{0}$	3.4810 × $10^{0}$	2.4614 × $10^{0}$
	176019	2.5007 × $10^{0}$	2.5049 × $10^{0}$	3.4308 × $10^{0}$	2.4541 × $10^{0}$
	196027	2.4710 × $10^{0}$	2.4669 × $10^{0}$	3.4666 × $10^{0}$	2.4373 × $10^{0}$
	208001	2.4531 × $10^{0}$	2.4979 × $10^{0}$	3.4256 × $10^{0}$	2.4431 × $10^{0}$
	209070	2.4784 × $10^{0}$	2.4959 × $10^{0}$	3.4292 × $10^{0}$	2.4287 × $10^{0}$
	288024	2.4695 × $10^{0}$	2.4674 × $10^{0}$	3.4295 × $10^{0}$	2.4190 × $10^{0}$
	296059	2.4581 × $10^{0}$	2.4693 × $10^{0}$	3.4487 × $10^{0}$	2.4253 × $10^{0}$
3	8068	2.8773 × $10^{0}$	2.8674 × $10^{0}$	3.8198 × $10^{0}$	2.8432 × $10^{0}$
	113044	2.8274 × $10^{0}$	2.8514 × $10^{0}$	3.7249 × $10^{0}$	2.7853 × $10^{0}$
	118035	2.8223 × $10^{0}$	2.8296 × $10^{0}$	3.7156 × $10^{0}$	2.7562 × $10^{0}$
	140075	2.8445 × $10^{0}$	2.8352 × $10^{0}$	3.8505 × $10^{0}$	2.7869 × $10^{0}$
	176019	2.8291 × $10^{0}$	2.8379 × $10^{0}$	3.8145 × $10^{0}$	2.7945 × $10^{0}$
	196027	2.7645 × $10^{0}$	2.7585 × $10^{0}$	3.7522 × $10^{0}$	2.7141 × $10^{0}$
	208001	2.8571 × $10^{0}$	2.8602 × $10^{0}$	3.8004 × $10^{0}$	2.7968 × $10^{0}$
	209070	2.8235 × $10^{0}$	2.8242 × $10^{0}$	3.8314 × $10^{0}$	2.7637 × $10^{0}$
	288024	2.8034 × $10^{0}$	2.7742 × $10^{0}$	3.8065 × $10^{0}$	2.7535 × $10^{0}$
	296059	2.7983 × $10^{0}$	2.8033 × $10^{0}$	3.7879 × $10^{0}$	2.7649 × $10^{0}$
4	8068	3.3991 × $10^{0}$	3.3858 × $10^{0}$	4.3397 × $10^{0}$	3.3712 × $10^{0}$
	113044	3.3266 × $10^{0}$	3.3666 × $10^{0}$	4.3057 × $10^{0}$	3.2873 × $10^{0}$
	118035	3.3603 × $10^{0}$	3.3396 × $10^{0}$	4.2313 × $10^{0}$	3.3014 × $10^{0}$
	140075	3.3783 × $10^{0}$	3.3943 × $10^{0}$	4.3459 × $10^{0}$	3.3260 × $10^{0}$
	176019	3.3373 × $10^{0}$	3.3555 × $10^{0}$	4.3529 × $10^{0}$	3.2856 × $10^{0}$
	196027	3.2157 × $10^{0}$	3.2376 × $10^{0}$	4.3401 × $10^{0}$	3.1716 × $10^{0}$
	208001	3.4067 × $10^{0}$	3.3868 × $10^{0}$	4.3219 × $10^{0}$	3.3447 × $10^{0}$
	209070	3.3971 × $10^{0}$	3.4012 × $10^{0}$	4.3750 × $10^{0}$	3.3325 × $10^{0}$
	288024	3.2882 × $10^{0}$	3.2859 × $10^{0}$	4.2860 × $10^{0}$	3.2241 × $10^{0}$
	296059	3.2707 × $10^{0}$	3.2861 × $10^{0}$	4.3621 × $10^{0}$	3.2193 × $10^{0}$
5	8068	3.9879 × $10^{0}$	3.8959 × $10^{0}$	4.7991 × $10^{0}$	3.9502 × $10^{0}$
	113044	3.9623 × $10^{0}$	4.0147 × $10^{0}$	4.6309 × $10^{0}$	3.8914 × $10^{0}$
	118035	3.8452 × $10^{0}$	3.8146 × $10^{0}$	4.7899 × $10^{0}$	3.7835 × $10^{0}$
	140075	3.8938 × $10^{0}$	3.9810 × $10^{0}$	4.7787 × $10^{0}$	3.8634 × $10^{0}$
	176019	4.0076 × $10^{0}$	4.0396 × $10^{0}$	4.8227 × $10^{0}$	3.8985 × $10^{0}$
	196027	3.7919 × $10^{0}$	3.7897 × $10^{0}$	4.6061 × $10^{0}$	3.7465 × $10^{0}$
	208001	4.1003 × $10^{0}$	4.0067 × $10^{0}$	4.9648 × $10^{0}$	3.9734 × $10^{0}$
	209070	3.9699 × $10^{0}$	3.9279 × $10^{0}$	4.7790 × $10^{0}$	3.8670 × $10^{0}$
	288024	3.8112 × $10^{0}$	3.8513 × $10^{0}$	5.0524 × $10^{0}$	3.8086 × $10^{0}$
	296059	3.8148 × $10^{0}$	3.8290 × $10^{0}$	4.9697 × $10^{0}$	3.7986 × $10^{0}$

## Data Availability

The original contributions presented in this study are included in the article. Further inquiries can be directed to the corresponding author.
